# The Harley Institute

**Published:** 1913-12-13

**Authors:** 


### THE HARLEY INSTITUTE.

### REFUSAL TO RENEW THE LICENCE.

At a meeting of the Public Control Committee of the London County Council on Friday, December 5, Mr. Alexander Stanley Flemmer applied for a licence for "The Harley Institute of Certificated Male and Female Nurses," Marylebone Road, as an employment agency for nurses. Several ladies objected to the application, and Mr. Hogg appeared on their behalf.

Mr. Hogg, in stating the case for the objectors, said that Mr. Flemmer advertised that anyone who went through a course of training at the Institute as a masseur would be guaranteed employment through his agency. The ground of his objection was that the employment agency was used to assist the applicant in running the training business, that the training business was not a genuine one for turning out properly qualified masseurs, that the applicant published misleading advertisements inducing people to go there, and after four weeks' training gave a high-sounding certificate; so that he was in effect turning out a large number of people who were incompetent, and that he was using the employment agency to enable him to do so. Mr. Hogg pointed out that there was an Incorporated Society of Trained Masseurs, and evidence would be given that the necessary course for massage was twelve to eighteen months, and he would also submit evidence to show that students had never even studied some of the things they were supposed to know. He read an advertisement recently issued by the applicant, which stated that three to ten pounds a week could be earned. In response to that advertisement a lady received a reply from the Harley Institute, in which were the following passages: "We do not recommend it (the Incorporated Society of Trained Masseurs), nor do we advise anyone to take its certificate. . . . Our certificate is recognised by the leading medical men and by the London County Council."

Dr. Lambert, a lady doctor practising in Cavendish Square, and an instructress in anatomy, who gave evidence, pointed out that a fully qualified masseur should receive instruction in anatomy, physiology, pathology, and other matters, which no person could possibly learn in one month.

Dr. Mina Dobbie gave evidence of a similar character, and added that although the National Hospital, Queen Square, gave a three-months' course, one of her friends had told her that even that period was inadequate. Mrs. A. E. Stewart, who stated that she carried on a school of Swedish exercises and massage in Marylebone Road, said that she took a certificated pupil from the Harley Institute, and afterwards discovered that the pupil lacked the necessary groundwork. Miss Victoria Kellaway said that she took the course of training at the Harley Institute, but after she had left she found that her knowledge was insufficient to cope with the work she was called upon to do, and she had since had additional instruction elsewhere. She only had five lessons in anatomy and none in pathology or physiology. The subjects practised on were men of eighteen to thirty years of age. She was unsuccessful in her efforts to get employment through the agency.

Mr. Flemmer gave evidence in support of his application. He stated that he had been trained at the National Hospital and privately in Sweden, and among the institutions where he had held appointments as a masseur or a dresser were the Royal Victoria Hospital, Netley, King's College Hospital, and St. George's Hospital. He gave the names of various medical men who had engaged pupils trained at the Harley Institute, including Sir Thomas Barlow and Sir Douglas Dawson, and he mentioned that Lord Stafford, Lord Kinnaird, and Lord Rocksavage were among those who had employed pupils from the Institute. He stated that pupils were admitted for an examination and were told that the training was unlimited, and that they could stay for two or three years if they liked. A person of ordinary competence could qualify for treating simple massage cases in three months under his system. Mr. Flemmer was shown a letter guaranteeing that a lady would be given training to deal with cases in one month. He replied that it was sent out without his knowledge, and he was surprised to see it. He believed that his course of instruction was better than that of the Incorporated Society of Trained Masseurs. He declared that the subjects used during the training were suitably clothed. Other evidence was called with the object of showing that the pupils could remain at the Institute as long as they liked without extra charge.

The Public Control Committee, after deliberation, refused to renew the employment agency licence.

